# Predictive value of plasma copeptin level for the risk and mortality of heart failure: a meta‐analysis

**DOI:** 10.1111/jcmm.13102

**Published:** 2017-02-28

**Authors:** Jian‐Jun Yan, Ying Lu, Zheng‐Ping Kuai, Yong‐Hong Yong

**Affiliations:** ^1^ Division of Cardiology Jiangning Hospital Affiliated Nanjing Medical University Nanjing China; ^2^ Department of Laboratory Medicine The First Affiliated Hospital of Nanjing Medical University Nanjing China; ^3^ Department of Cardiology Shanghai Meishan Hospital of Nanjing Medical University Nanjing China; ^4^ Department of Cardiology the First Affiliated Hospital of Nanjing Medical University Nanjing China

**Keywords:** copeptin, heart failure, meta‐analysis

## Abstract

Epidemiologic studies are inconsistent regarding the association between plasma copeptin level and heart failure (HF). The aim of this study was to perform a meta‐analysis to determine whether high level of copeptin is correlated with incidence of HF and mortality in patients with HF. We searched PUBMED and EMBASE databases for studies conducted from 1966 through May 2016 to identify studies reporting hazard ratio (HR) estimates with 95% confidence intervals (CIs) for the association between plasma copeptin level and HF. A random‐effects model was used to combine study‐specific risk estimates. A total of 13 studies were included in the meta‐analysis, with five studies on the incidence of HF and eight studies on the mortality of patients with HF. For incidence of HF, the summary HR indicated a borderline positive association of high plasma copeptin level with HF risk (HR, 1.60; 95% CI, 0.90–2.85). Furthermore, an increase of 1 standard deviation in log copeptin level was associated with a 17% increase in the risk of incident HF (HR, 1.17; 95% CI, 1.02–1.33). For all‐cause mortality of patients with HF, we also found a significant association between elevated plasma copeptin level and increased mortality of HF (HR, 1.76; 95% CI, 1.33–2.33). Our dose–response analysis indicated that an increment in copeptin level of 1 pmol/l was associated with a 3% increase in all‐cause mortality (HR, 1.03; 95% CI, 1.01–1.05). In conclusion, our results suggest that elevated plasma copeptin level is associated with an increased risk of HF and all‐cause mortality in patients with HF.

## Introduction

Arginine vasopressin (AVP), also known as antidiuretic hormone, is synthesized in the hypothalamus and secreted from the neurohypophysis in response to haemodynamic and osmotic changes 1. Circulating level of AVP increases in various pathological conditions, especially in cardiovascular diseases 2. However, circulating AVP is not a useful biomarker due to its short half‐life and instability 3. Copeptin, the C‐terminal part of the pro‐AVP, is secreted in parallel with AVP during processing of the precursor peptide. In contrast to AVP, copeptin is more stable and easier to measure with an automated immunoassay, making it potentially suitable as a surrogate biomarker for the unstable AVP 4.

Heart failure (HF) is a complex clinical syndrome characterized by increased activation of neuroendocrine axis 5. To date, several studies have been conducted to investigate the association of plasma copeptin level with the risk and prognosis of HF 6. However, the results of those observational studies remain inconsistent and have not yet been quantitatively summarized. Hence, we chose to conduct a meta‐analysis to combine the results from the available prospective studies, to evaluate whether high plasma level of copeptin is associated with incidence of HF and adverse outcome in patients with HF.

## Methods

### Search strategy

We searched the PUBMED and EMBASE databases that included the years 1966 through May 2016. We used search terms ‘copeptin’ or ‘CT‐proAVP’ or ‘CT‐pro vasopressin’, combined with ‘heart failure’ or ‘cardiac failure’ or ‘AHF’ or ‘CHF’ in the full‐text option. Titles and abstracts were screened to exclude any obvious irrelevant studies. References of relevant studies and review articles were checked for additional studies. Two authors (J.J.Y and Y.L) conducted all searches independently.

### Eligibility criteria

Studies were included if they met the following criteria: (*i*) prospective cohort studies of adult patients with longer than 1 year of follow‐up and with a sample size of at least 100 patients; (*ii*) the outcome of interest was total HF incidence or mortality; (*iii*) hazard ratio (HR) estimates with corresponding 95% confidence intervals (CIs) were reported. If data were duplicated in more than one study, the most recent study was eligible for inclusion.

### Data extraction

The following data were extracted by two investigators (J.J.Y and Y.L) independently using a predefined data extraction form the first author's name; publication year; country; sex; age; number of participants; years of follow‐up; outcome; the HR and corresponding 95% CI; and adjustment variables. For each study, we extracted the risk estimates that reflected the greatest degree of control for potential confounders. The results were compared, and any discrepancies were resolved by consensus.

### Quality assessment

We chose to use the Newcastle–Ottawa Scale 7 for quality assessment because this tool appropriately evaluates the three most important domains of prospective cohort studies: selection of study participants (0–4 points), comparability of populations (0–2 points) and ascertainment of exposure to risk (0–3 points). Ratings for each item were added to provide a study quality score (maximal score = 9). Disagreements were resolved by discussion between the reviewers or in consultation with a third reviewer.

### Statistical analysis

Analyses were performed using the Stata 12.0 software package (STATA Corp, College Station, TX, USA). The measure of effect of interest was HR with the corresponding 95% CI. Study‐specific risk estimates were extracted from each article, and log risk estimates were weighted by the inverse of their variances to obtain a pooled risk estimate. Studies were combined using the DerSimonian and Laird random‐effects model, which considers both within‐ and between‐study variations 8. The likelihood of heterogeneity of the studies was assessed using the *Q* and *I* ^2^ statistics. To avoid type II errors resulting from low power, we set the significance level at 0.10 instead of the more conventional level of 0.05 9. Subgroup analysis and sensitivity analysis were performed to analyse the potential sources of heterogeneity. For dose–response analysis, we used the generalized least‐squares trend estimation (GLST) analysis based on the methods developed by Greenland and Longnecker 10, 11 to estimate study‐specific slopes from the natural logarithm of the HR across categories of exposure. For each study, the median or mean level of copeptin for each category was assigned to each corresponding HR estimate. When the median or mean of per category was not provided in the article, we assigned to each class the dose corresponding to the mid‐point of upper and lower boundaries. For the open‐ended categories, we estimated the median value assuming a normal distribution density function. In studies that did not provide the number of cases and person‐years in each exposure category, the variance‐weighted least square (VWLS) regression model was used to estimate the slopes. Because these two methods require the risk estimates with their variance estimates for at least three quantitative exposure categories, the studies with only two categories were not included in this analysis. Then, we obtained the summary HR estimates by pooling the study‐specific slopes, using the inverse of the corresponding variances as weights. Finally, publication bias was evaluated with Egger's regression asymmetry test in which a *P* value less than 0.10 was considered statistically significant 12.

## Results

### Literature search and study characteristics

**Figure 1:**
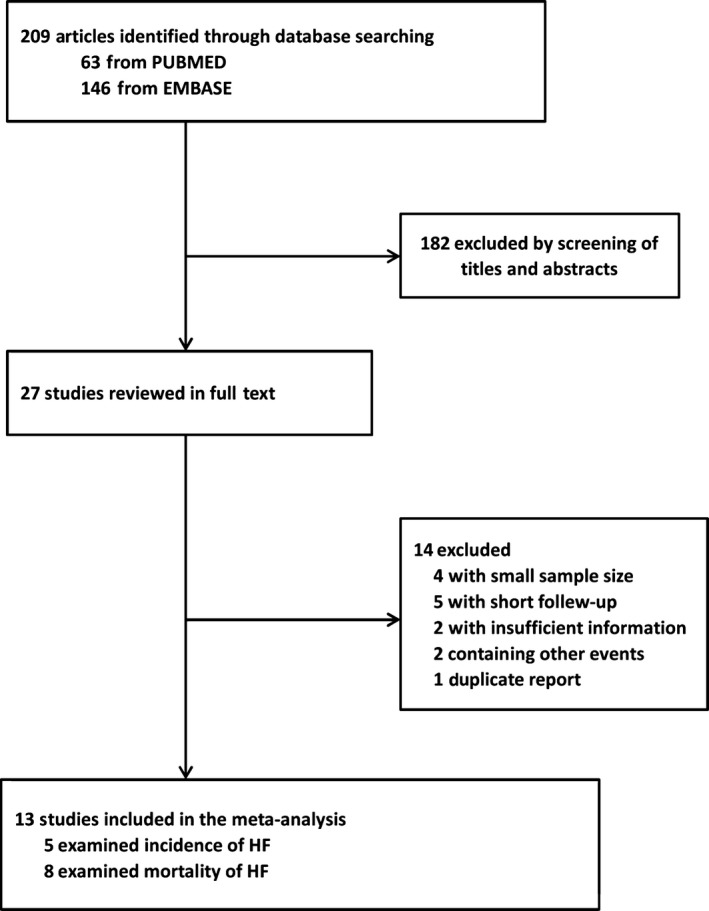
Flow chart for indentifying eligible studies.

As shown in Figure 1, after review of 209 studies, our literature search identified 27 potentially relevant studies concerning the correlation of copeptin level with HF. Nine articles were excluded because of small sample size (<100 patients) 13, 14, 15, 16 or short period of follow‐up (<1 year) 17, 18, 19, 20, 21. Two reports 22, 23 were excluded because of insufficient information. Another two studies 24, 25 were excluded because the end‐point contained other cardiovascular events. One study 26 was excluded because it was updated by the same author 27 in 2010. Thus, our meta‐analysis on the correlation of copeptin level with HF included 13 papers in total, with five studies 28, 29, 30, 31, 32 on the risk of HF and eight studies 27, 33, 34, 35, 36, 37, 38, 39 on the mortality of HF.

Table 1 summarizes the baseline patient characteristics and design of the five studies on the association of copeptin level with incident HF. A total of 15,631 participants and 629 cases were included. Two studies 29, 31 enrolled community‐based populations, and the other three 28, 30, 32 studied patients with coronary artery disease (CAD). Two Studies were conducted in the United States 30, 32, and the remaining three were in Germany 28, Sweden 29 and the United Kingdom 31, respectively. The follow‐up duration ranged from 1 to 14 years. All studies adjusted covariates which may affect the association of copeptin level with the risk of HF.

**Table 1:** Characteristics of studies on plasma copeptin level and incidence of HF

Study (author, year, ref)	Area	Study design	Study population	Age (years)	Male (%)	Follow‐up	Incident HF (n)	HF definition	Sample	Assay kit	Comparison copeptin level	HR (95% CI)	Adjustments	Quality score
Kelly *et al*., 2008 28	Germany	Cohort	274 patients with acute MI	63 (24–91)	75	Median = 381 days	30	Medical records of treatment for clinical HF with high‐dose diuretics, inotropes or intravenous nitrate	Plasma	BRAHMS	Continuous	3.01 (1.10–8.21)	Age, creatinine, anterior, DM, hypertension, previous angina, LVEF, heart rate, Killip class	4/2/2
Smith *et al*., 2010 29	Sweden	Cohort	5187 residents	57.6 ± 5.9	41	Median = 13.8 years	112	ICD‐8, 9, 10	Plasma	BRAHMS	Per 1 S.D. log copeptin increase	1.35 (1.03–1.77)	Age, gender, BP, antihypertensive treatment, BMI, LDL, HDL, smoking, DM, history of MI	3/2/3
Sabatine *et al*., 2012 30	United States of America	Cohort	1868 patients with stable CAD	64.1 ± 8.2	82.1	Median = 4.8 years	56	Medical records of hospitalization with a primary cause of HF	Plasma	BRAHMS	Per 1 S.D. log copeptin increase	1.08 (0.83–1.40)	Age, gender, weight, hypertension, DM, smoking, prior MI, prior PCI or CABG, systolic BP, eGFR, ratio of apoB/apoA, LVEF, aspirin use, beta‐blocker use, lipid‐lowering medication use	4/1/2
Wannamethee *et al*., 2014 31	United Kingdom	Cohort	3870 men with no diagnosed HF	68.61 ± 5.51	100	Mean = 11 years	254	Twice medical records of HF symptoms, signs, investigations and treatment response	Plasma	BRAHMS	Per 1 S.D. log copeptin increase	1.13 (0.97–1.42)	Age, smoking, physical activity, social class, alcohol intake, left ventricular hypertrophy, systolic BP, antihypertensive drugs, history of MI, angina, eGFR, FEV1, albumin, CRP	4/2/3
											1st quartile (<2.46 pmol/l)	1		
											2nd quartile (2.46–3.85 pmol/l)	1.03 (0.68–1.55)		
											3rd quartile (3.86–6.32 pmol/l)	1.17 (0.79–1.73)		
											4th quartile (>6.32 pmol/l)	1.18 (0.79–1.76)		
O'Malley *et al*., 2014 32	United States of America	Cohort	4432 patients with non‐ST‐elevated ACS	NM	64.7	1 year	177	Medical records of HF symptoms, signs and treatment response	Plasma	BRAHMS	4th quartile vs. 1st–3rd quartile	2.12 (1.55–2.89)	Age, CAD, CAD risk factors, repeated rest pain, aspirin use, ST depression, history of chronic HF, creatinine clearance, BNP, cTnI	3/0/2

ACS, acute coronary syndrome; BMI, body mass index; BNP, brain natriuretic protein; BP, blood pressure; CABG, coronary artery bypass graft; CAD, coronary artery disease; CI, confidence interval; CRP, C‐reactive protein; CVD, cardiovascular disease; DM, diabetes mellitus; eGFR, estimated glomerular filtration rate; FEV1, forced expiratory volume in one‐second; HDL, high‐density lipoprotein; HF, heart failure; HR, hazard ratio; ICD, International Classification of Diseases; LDL, low‐density lipoprotein; LVEF, left ventricular ejection fraction; MI, myocardial infarction; PCI, percutaneous coronary intervention; S.D., standard deviation.

Table 2 summarizes the main features of the eight studies on the predictive value of copeptin level for all‐cause mortality in patients with HF. A total of 2824 patients with chronic HF and 361 with acute HF were included. Three studies 27, 34, 35 were conducted in Austria, two studies 37, 38 were in Denmark, and the remaining three studies were in Norway 36, Italy 39 and Sweden^33^, respectively. The follow‐up duration ranged from 1 to 13 years. All studies, except Tentzeris *et al*.'s 35, adjusted the impact of confounders when assessing the correlation between copeptin level and prognosis of HF.

**Table 2:** Characteristics of studies on plasma copeptin level and all‐cause mortality of patients with HF

Study (author, year, ref)	Area	Study design	Study population	Age (years)	Male (%)	Follow ‐up	Mortality (n)	Sample	Assay kit	Comparison copeptin level	HR (95% CI)	Adjustments	Quality score
Gegenhuber *et al*., 2007 34	Austria	Cohort	137 patients with acute destabilized HF	75 (65–80) for alive, 79 (72–83) for dead	93.4	1 year	41 death	Plasma	BRAHMS	1st tertile (<15 pmol/l)	1	Unadjusted	4/2/2
										2nd tertile (15–45 pmol/l)	2.22 (1.89–3.60)		
										3rd tertile (>45 pmol/l)	5.38 (3.51–9.12)		
										3rd tertile (>45 pmol/l) vs. 1st–2nd tertile (<45 pmol/l)	2.26 (1.11–4.62)	Age, systolic BP, renal dysfunction, systolic dysfunction, NYHA Classes	
Voors *et al*., 2009 36	Norway	Cohort	224 patients who developed acute HF after acute MI	68 ± 10	70.0	33 ± 7 months	32 death	Plasma	BRAHMS	Per doubling	1.83 (1.26–2.64)	Age, gender, renal function, previous MI, DM, treatment group	4/2/2
Masson *et al*., 2010 39	Italy	Cohort	1237 patients with chronic and stable HF	67 ± 11	80.4	Median = 3.9 (3.1–4.6) years	332 death	Plasma	BRAHMS	1st tertile (0.15–9.6 pmol/l)	1	Age, BMI, NYHA class, LVEF, ischaemic aetiology of HF, eGFR, heart rate, BP, diabetes, atrial fibrillation, COPD, prescription of beta‐blockers, diuretics or digitalis, serum concentrations of bilirubin and fibrinogen	4/1/2
										2nd tertile (9.61–19.1 pmol/l)	0.99 (0.66–1.31)		
										3rd tertile (19.2–22.8 pmol/l)	1.52 (1.12–2.07)		
Neuhold *et al*., 2010 27	Austria	Cohort	181 patients with chronic systolic HF	70 ± 12	65.0	2 years	36 death	Plasma	BRAHMS	Continuous	1.93 (1.23–3.01)	Age, gender, GFR, DM, ischaemic aetiology of HF	4/2/2
Tentzeris *et al*., 2011 35	Austria	Cohort	172 consecutive patients with stable chronic HF	65.87 ± 12.18	77	Median = 1301 (707–1636) days	36 death	Plasma	BRAHMS	>16.4 pmol/l	2.69 (1.61–4.50)	Unadjusted	4/0/2
Alehagen *et al*., 2011 33	Sweden	Cohort	470 elderly patients with symptoms of HF	73	52.1	Median = 4725 (242–5112) days	226 all‐cause death, including 146 cardiovascular event death	Plasma	BRAHMS	1st quartile (<5.70 pmol/l)	1	Gender, DM, haemoglobin, GFR, NYHA class, ischaemic heart disease, hypertension, LVEF	4/2/3
										2nd quartile (5.70–9.95 pmol/l)	1.04 (0.72–1.51)		
										3rd quartile (9.96–18.0 pmol/l)	1.33 (0.76–2.33)		
										4th quartile (>18.0 pmol/l)	2.04 (1.38–3.02)		
Balling *et al*., 2012 37	Denmark	Cohort	340 HF patients with left ventricular systolic dysfunction	71.3 ± 10.8	72.9	Median = 55 months	165 death	Plasma	BRAHMS	3rd tertile (>22.5 pmol/l) vs 1st (<11.5 pmol/l) + 2nd (11.5–22.5 pmol/l) tertile	1.2 (0.9–1.8)	Age, gender, ischaemic heart disease, systolic BP, heart rate, sodium, eGFR, LVEF, loop diuretic dose, NYHA class, NT‐proBNP	4/0/2
Bosselmann *et al*., 2013 38	Denmark	Cohort	424 patients with systolic HF	72	71.0	Median = 4.5 (2–7.7) years	252 death	Plasma	BRAHMS	Continuous	1.02 (1.01–1.04)	Age, gender, NYHA class, LVEF, DM, ischaemic heart disease, eGFR	4/0/3

BNP, brain natriuretic protein; BMI, body mass index; BP, blood pressure; CI, confidence interval; COPD, chronic obstructive pulmonary disease; DM, diabetes mellitus; eGFR, estimated glomerular filtration rate; HF, heart failure; HR, hazard ratio; LVEF, left ventricular ejection fraction; MI, myocardial infarction; NYHA, New York Heart Association.

### Plasma copeptin level and the risk of HF

**Figure 2:**
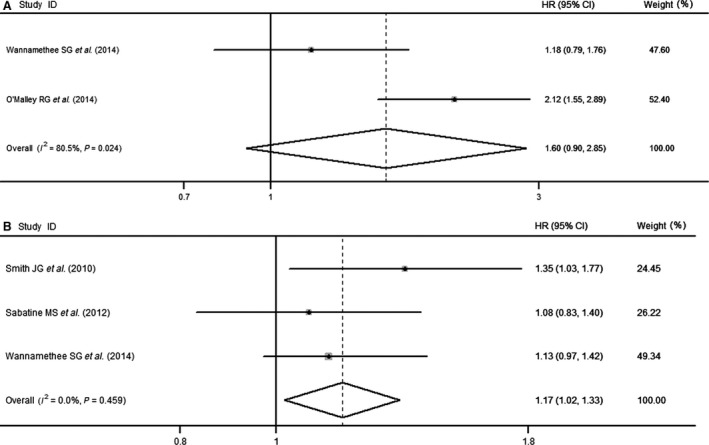
(**A**) Forest plot demonstrating the association of plasma copeptin level with incidence of heart failure (HF; categorical variable). (**B**) Forest plot demonstrating the association of plasma copeptin level with incidence of HF (continuous variable). CI, confidence interval; HR, hazard ratio.

Among the five studies, one 31 reported copeptin level as both categorical and continuous variables, one 32 reported as categorical variable, and three 28, 29, 30 reported as continuous variables. When copeptin level was treated as categorical variable, the pooled HR based on two studies was 1.60 (95% CI, 0.90–2.85) (Fig. 2A). When copeptin level was treated as continuous variable, there was a dose–effect relationship between copeptin level and incidence of HF. For each 1 standard deviation (S.D.) increase in log copeptin level, the overall pooled HR for incident HF was 1.17 (95% CI, 1.02–1.33) (Fig. 2B). The Egger's test for publication bias was not statistically significant (*P* = 0.706).

### Plasma copeptin level and all‐cause mortality of HF

**Figure 3:**
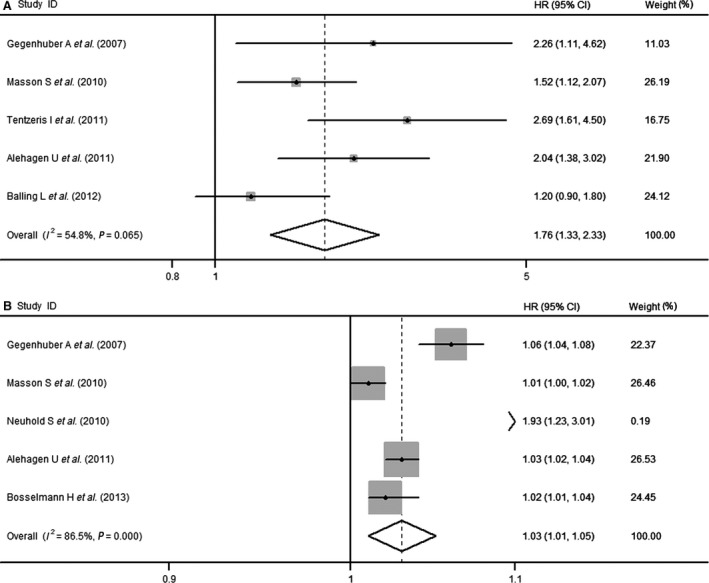
(**A**) Forest plot demonstrating the association between plasma copeptin level and all‐cause mortality in patients with heart failure (HF) (categorical variable). (**B**) Forest plot demonstrating the dose–effect relationship between plasma copeptin level and all‐cause mortality in patients with HF. CI, confidence interval; HR, hazard ratio.

The pooled HR from five studies 33, 34, 35, 37, 39 that treated copeptin level as categorical variables was 1.76 (95% CI, 1.33–2.33) for all‐cause mortality, with mild heterogeneity (*I* ^2^ = 54.8%, *P* = 0.065) (Fig. 3A). We used subgroup analyses and sensitivity analysis to identify sources of heterogeneity. Stratified analysis by geographical region, gender, age and sample size is shown in Table 3. When it was conducted by geographical region, the predictive effect of copeptin for the all‐cause mortality of heart failure was more significant in Austrians with no significant heterogeneity (HR, 2.53; 95% CI, 1.67–3.85; *P* for heterogeneity = 0.698; *I* ^2^ = 0.0%) than in people from other countries. When it was conducted by sex, no significant heterogeneity was observed in males (HR, 1.62; 95% CI, 1.22–2.15; P for heterogeneity = 0.317; *I* ^2^ = 0.3%). The result of sensitivity analysis in which one study was removed at a time showed that most of heterogeneity was accounted for the study by Balling *et al*. 37. After excluding this single study, there was no study heterogeneity (*P* = 0.343, *I* ^2^ = 9.9%), and the HR was 1.75 (95% CI, 1.37–2.14). No potential publication bias was found (Egger's test: *P* = 0.198).

**Table 3:** Subgroup analyses of the association between plasma copeptin level and all‐cause mortality in patients with HF

Subgroup	Number	HR (95% CI)	Heterogeneity test		
			*Q*	*P*	*I* ^2^ (%)
Geographical region					
Austria	2	2.53 (1.67–3.85)	0.15	0.698	0.0
Other countries	3	1.53 (1.16–2.03)	3.96	0.138	49.4
Males					
>80%	2	1.62 (1.22–2.15)	1.00	0.317	0.3
<80%	3	1.82 (1.14–2.92)	7.78	0.020	74.3
Age					
<70	2	1.95 (1.12–3.39)	3.49	0.062	71.4
≥ 70	3	1.68 (1.11–2.55)	2.48	0.081	60.2
Sample size					
<400	3	1.87 (1.06–3.29)	7.46	0.024	73.2
>400	2	1.72 (1.29–2.28)	1.34	0.247	25.5

CI, confidence interval; HF, heart failure; HR, hazard ratio.

Our dose–response analysis indicated that an increase in copeptin level of 1 pmol/l was associated with a 3% increase in all‐cause mortality (HR, 1.03; 95% CI, 1.01–1.05; Fig. 3B). There was a statistically significant heterogeneity among the study results (*P* = 0.000; *I* ^2^ = 86.5%). The sensitivity analysis showed that most of heterogeneity was accounted for the study by Neuhold *et al*. 27. After excluding this single study, there was no study heterogeneity (*P* = 0.761, *I* ^2^ = 0.0%), and the HR for 1 pmol/l increase was 1.01 (95% CI, 1.00–1.02).

## Discussion

The present meta‐analysis evaluated the association of plasma copeptin level with the risk and mortality of HF based on published results from 13 cohort studies. Our results demonstrate an equivocal positive association between elevated plasma copeptin level and increased risk of suffering from HF and suggest that higher plasma level of copeptin was also a predictor of all‐cause mortality in patients with HF.

Some studies 13, 16, 21, 23 which were not included in our meta‐analysis also demonstrated significant positive correlation between elevated plasma copeptin level and incidence of HF. In a prospective single‐hospital study based on patients with post‐acute myocardial infarction (AMI), Khan *et al*. 23 found that plasma copeptin level was a significant independent predictor of HF. Another two study conducted by Reinstadler *et al*. 13, 16 also showed that plasma copeptin level could be a predictor for the risk of adverse cardiac remodelling after AMI. In a study of patients presenting to the emergency department with dyspnoea as the chief complaint, plasma level of copeptin was significantly higher in patients with acute HF when compared to those with dyspnoea attributable to other causes 21. In our meta‐analysis, we observed a borderline positive association between elevated plasma copeptin level and the risk of HF (HR, 1.60; 95% CI, 0.90–2.85) when copeptin level was treated as categorical variable. Moreover, the pooled results when copeptin level was treated as continuous variable revealed that an increase of 1 S.D. increase in log copeptin level was related to a significant 17% increase in the risk of HF without indication of heterogeneity (*P* = 0.459, *I* ^2^ = 0.00%). However, this may be an overestimate of the true magnitude of the association because of the limited number of studies. Two studies in which larger proportion of males (>80%) were recruited showed no significant positive relation 30, 31, whereas it has been demonstrated that copeptin levels were higher in the male volunteers compared with female 40. Thus, the effects of gender differences on the association of copeptin with HF must be of concern and still warrant further investigation. Furthermore, in healthy individuals, the median value of plasma copeptin has been found to be 4.2 pmol/l (range, 1–13.8 pmol/l) 4. Considering the observed plasma copeptin levels in patients with HF (10–50 pmol/l) 41 were much higher than 4.2 pmol/l, copeptin may also be served as a novel biomarker for the diagnosis of HF. However, in two recent diagnostic studies based on older residents, although plasma copeptin levels were elevated in patients with acute HF, using copeptin did not significantly improve the diagnosis of HF 42, 43. It seems that copeptin can provide important prognostic information for the development of HF in patients with CAD, especially in patients with previous MI; however, its diagnostic role in HF has not been convinced. Studies with larger sample sizes are needed to validate the value of copeptin assessment alone or combined with other biomarkers, such as brain natriuretic protein (BNP), for rapid rule out of HF.

Another important finding of our meta‐analysis is that elevated plasma copeptin level was associated with significantly increased all‐cause mortality in patients with HF. In 2006, copeptin was first demonstrated to be an excellent predictor of outcome in advanced HF patients by Stoiser *et al*. 22. With a mean follow‐up period of 15.8 months, copeptin was found to be superior to BNP in predicting mortality and combined end‐point of mortality and rehospitalization due to HF. Neuhold *et al*. 26 also found that copeptin was the most potent single predictor of mortality in HF patients with New York Heart Association (NYHA) functional class II and III. In NYHA functional class IV, although sodium level was the best predictor of mortality, copeptin could still add additional independent information in contrast to BNP. Similarly, for the prediction of 90‐day mortality in patients with acute HF, copeptin was also an independent predictor of mortality with additive prognostic value 17, 20. The data of our meta‐analysis are consistent with the results of above studies. In subgroup analyses by geographical region, gender, age and sample size, we found that the positive correlation between them was generally unchanged. Our finding was further strengthened by the absence of publication bias and the result of dose–response analysis, which indicates that an increase of 1 pmol/l of copeptin level was related to a 3% increase in all‐cause mortality of patients with HF. It appears that copeptin could be served as a novel superior predictor of outcome in HF besides BNP. However, in the study of Balling *et al*.^37^, although copeptin was a significant predictor of hospitalization or death (HR, 1.4; 95% CI, 1.1–1.9), it did not predict mortality alone independently from NT‐proBNP. Indeed, on the basis of our sensitivity analysis, after exclusion of this study, we found a more significantly positive association (HR, 1.94; 95% CI, 1.50–2.51) with more homogeneous (*P* = 0.248; *I* ^2^ = 27.4%). The discrepancy may be explained in part by the more serious status of patients in this study, which contained a large proportion of patients with NYHA class III/IV 37. Therefore, larger studies are needed to further investigate the different predictive abilities of copeptin and NT‐proBNP for patients with HF, especially for patients in different stages. Considering HF is a dynamic syndrome characterized by dramatically increased neurohormonal activation, it should be better to use the combination of copeptin with other biomarkers to improve the predictive effect of adverse outcome in patients with HF.

The mechanisms by which copeptin is elevated in patients with HF and predicts worse prognosis remain unclear. As AVP secretion is partly stimulated in response to increased osmolality, it seems unexpected that AVP is increased in patients with HF, which is characterized with low osmolality. The potential explanation may attribute to hyponatraemia and decreased cardiac output, which can also activate the secretion of AVP through baroreceptors 44. This elevation might be beneficial to maintain blood pressure in the short term, but long‐term excessive secretion of AVP can lead to adverse cardiac remodelling process via vasopressin 1a receptors 45. However, AVP has a short half‐life of 24 minutes 3. Thus, copeptin might better reflect stable levels of AVP related to the severity of HF.

Several limitations of our study should be considered. First, the articles included were all published in English; limited resources prevented us from including articles published in other languages. Second, the number of included studies is relatively small. Although the Egger's test was not statistically significant, publication bias may still exist. Third, we cannot resolve uncontrolled confounders as a potential explanation for the observed association, because a meta‐analysis cannot exclude residual or unknown confounders that could be inherent in those original studies. Fourth, all studies included were conducted in Europe and the United States; therefore, the results should be extrapolated to all populations with caution.

## Conclusions

In conclusion, results from the present study suggest that elevated plasma copeptin level is associated with an increased risk of HF and all‐cause mortality in patients with HF. Copeptin may serve as a practical guide for the prevention and treatment of HF.

## Funding source

This work was supported by grants from the Six Talent Peaks Project in Jiangsu Province (No. WSN‐037) to Y.H.Y.

## Conflict of interest

The authors declare that they have no competing interest.
